# Effects of a One Year Intensive Multidisciplinary Rehabilitation Program for Patients with Huntington’s Disease: a Prospective Intervention Study

**DOI:** 10.1371/currents.hd.9504af71e0d1f87830c25c394be47027

**Published:** 2013-09-20

**Authors:** Anu Piira, Marleen R. van Walsem, Geir Mikalsen, Kjell Haavik Nilsen, Synnove Knutsen, Jan C. Frich

**Affiliations:** Department of Research and Development, North Norway Rehabilitation Center, Tromsø, Norway; Department of Neurohabilitation, Division of Surgery and Clinical Neuroscience, Oslo University Hospital, Oslo, Norway; Department of Research and Development, North Norway Rehabilitation Center, Tromsø, Norway; Vikersund Kurbad Rehabiltation Centre, Vikersund, Norway; Department of Research and Development, North Norway Rehabilitation Center, Tromsø, Norway; ProfessorUniversity of Oslo

## Abstract

Objective: To assess the effects of an intensive, multidisciplinary rehabilitation program for patients with early to mid-stage Huntington’s disease. Design: A prospective intervention study. Setting: Two Norwegian inpatient rehabilitation centers. Subjects: 37 patients, with early- to midstage Huntington’s disease Interventions: A one year rehabilitation program, consisting of three admissions of three weeks each, and a five-day evaluation stay approximately 3 months after the last rehabilitation admission. Focus was on physical exercise, social activities, and group/teaching sessions. There was also emphasis to implement of coordinated health care and social services for the patients. Main outcome measures: standard measures for motor function, including gait and balance, cognitive function, including MMSE and UHDRS cognitive assessment, anxiety and depression, activities of daily living (ADL), health related quality of life and Body Mass Index (BMI). Results: Significant improvements were observed in gait function, balance, in physical quality of life, anxiety and depression, as well as in BMI. ADL-function remained stable with no significant decline. Only one cognitive measure (SDMT) showed significant decline, while no decline was observed for the remaining cognitive measures. Conclusion: A multidisciplinary intensive rehabilitation program in patients with early and mid stage HD is associated with improved balance, gait function, physical quality of life and with reduced depressive and anxiety symptoms. Longer follow-up is needed to assess if these positive effects are sustained. There should be emphasis to establishment of long term and coordinated health care services for the HD patient

## Introduction

Huntington’s disease (HD) is a hereditary autosomal neurodegenerative disorder caused by an expanded Cytosine-Adenine-Guanine (CAG) repeat in the HD gene 1 . The disease is characterized by motor disturbances, psychiatric symptoms and cognitive decline 2 ^,^ 3. A clinical diagnosis of HD is typically made when an individual has overt motor symptoms and a family history of Huntington’s disease. Average age of diagnosis is 40-45 years, but often symptoms have already been present for several years at the time of diagnosis 2. Disease duration is commonly between 15-20 years, but symptom development and severity vary greatly between individuals. Motor symptoms are most visible and result in gait and balance problems. However, cognitive and behavioral changes are known to frequently occur many years before clinical diagnosis 4 ^,^ 5 resulting in functional decline already early in the disease 6. Additionally, metabolic changes (increased appetite, weight loss etc.) and sleep disturbances are known to develop 4 ^,^ 7 ^,^ 8. Current treatment of persons with HD consists mainly of symptom management and improving quality of life 7. A multidisciplinary approach in the management of persons with HD is recommended 9.

In recent years, the interest in investigating the effects of non-therapeutic agents for managing and improving the symptoms of HD has been growing. Multiple studies have investigated treatment with physiotherapy. These studies showed beneficial effects and have also investigated sensitive standard physiotherapy outcome measures 10 ^-^ 15. Additionally, two important multidisciplinary rehabilitation studies have been conducted 16 ^-^ 18. Zinzi et al (2007) reported that early to mid-stage HD patients who participated in a two-year intensive multidisciplinary rehabilitation program, containing six in-patient stays of three weeks in a rehabilitation center, were able to maintain or improve their cognitive and motor function 16. However, only 11 of 40 participants completed the full two-year program. Reasons for loss-to-follow-up are unknown. Participants and caregiver´s who completed at least one course of the full rehabilitation protocol program (3-week intensive multidisciplinary treatment) reported improvement in physical function, swallowing, balance, increased independence, improvement in mood, less apathy and improvement in social relations 17. Another study is a pilot study comparing participation in a multidisciplinary rehabilitation program (once a week over 9 months combined with home-based exercises three times a week for 6 months) with a control group of early to middle-stage HD patients. The results of the study suggest therapeutic benefit and good tolerance of multidisciplinary rehabilitation 18. Currently, there still is a lack of randomized clinical trials investigating the effects of multidisciplinary rehabilitation programs or other forms of non-medical treatments.

In 2009, The Norwegian Directorate of Health initiated the establishment of a pilot project to investigate effects of HD patients’ participation in an intensive multidisciplinary rehabilitation program. The primary aim of the project was to replicate the results reported by Zinzi et al. (2007). Additionally, the present project aimed at performing a more in depth quantitative evaluation by using a greater variety of assessments, including measures of quality of life, ADL function, motor and cognitive function throughout three times 3-week intensive multidisciplinary rehabilitation program. Another goal was to obtain better retention numbers by closely following up the participants during the course of the program. Providing information about the rehabilitation program prior to the first time admission and information in the end of each rehabilitation stay for participants and his/hers family member and local caregivers were important to secure retention in programs. Participants/ families had also possibility to contact directly institutions also between the rehabilitation stays. The final and important goal of this project was to implement the establishment or initiation of coordinated health care and social services for participants, using a so called “Individual plan”. An Individual Plan is a statutory tool for co-operation between patient and local health care providers or the labour and welfare organization to secure long-term follow-up for persons with chronic diseases and disabilities in Norway. It contains an outline of patient’s goals, recourses and the services he or she may require due to disability. It is not conditional on any particular diagnosis or age. It’s not required that patient need to receive specialist healthcare to receive this plan and it can be used at any level of health care services. It also specifies when the different actions are to be carried out and who is responsible to execute these actions 19.

This project included a quantitative and a qualitative evaluation. The present paper reports the quantitative results after one-year participation in the program. Results of the qualitative evaluation of the experiences of the participants, their family members and healthcare providers will be reported elsewhere.

## Methods

**Subjects**

A total of 37 patients were enrolled in the rehabilitation program in the two rehabilitation centers in Vikersund and Tromsø. The following inclusion criteria were used: 1) age >18 years, 2) known genetic diagnosis of Huntington´s disease, 3) early to mid stage HD, equivalent to stages I-III on Shoulson & Fahn rating scale, 4) no diagnoses of severe psychiatric disease and 5) no apparent severe impairment in general cognitive function at the time of first admission.

There was no a specific cognitive testing prior to patient enrollment to the program. The referring physicians were asked to do a clinical evaluation of the patient, and the main focus was whether or not the patient was able to stay in the institution within being helped with daily functions such as dressing/undressing and daily hygiene.

**Procedures**

Information about the project was spread by posting information on the web sites of both inpatient rehabilitation centers, the web site of a specialized national competence center for rare diseases, and announcements through the Norwegian patient association for HD. Participants were referred by their general practitioners or by specialists in neurology or psychiatry. Based on referrals and the patients’ own preference, they were enrolled in the rehabilitation programs in the two sites. The project was submitted to the ethics committee who considered that a formal approval was not necessary (ref. 2010/2629-7), and thus approval was only obtained from the Norwegian Social Science Data Services. Participants were included in groups of four to six persons during 2010 – 2012. All participants and their family members received written and oral information, and gave their written informed consent to participate in the project. The participants in the present study all completed an evaluation stay three months after discharge of the third stay. For all participants the following demographic information was collected from the medical records at the time of the first admission: age, gender, marital status, estimated disease duration. Additionally, baseline clinical characteristics were collected using standardized assessments, including the motor, functional and behavioral assessment of Unified Huntington´s Disease Rating Scale (UHDRS) 20.

**Description of the Rehabilitation program.**

The structure of the rehabilitation program was identical for both rehabilitation centers, with three in-patient stays of three weeks each during one year. The program consisted of up to 8 hours of various activities five days a week, from Monday to Friday, and one of the sites (Tromsø) also offered four hours of supervised activities during the weekend. Each day included specific daily training activities with physio-, occupational- and speech therapists, as well as training in groups in the gym and/or in a swimming pool. Additionally, there were patient education sessions and group discussions for participants. Physical therapy focused on improvement of balance and gait, occupational therapy sessions included training of Activities of Daily Living (ADL) and cognitive function, fine motor exercises and assessment of the need for assistive devices. Dietitians followed-up each participant and a social worker and psychologist also offered individual follow-up. For those who had a low Body Mass Index (BMI) (< 21) and / or had problems with swallowing, dietary adjustments were made and a dietitian monitored their progress during their stays. Nurses observed and helped participants with reduced cognitive function or who showed problems in ADL function due to chorea.

If necessary, participants received medication adjustment throughout the project period. Most adjustments were made by a neurologist in order to reduce choreatic movements or other motor and clinical symptoms such as depressive symptoms and sleep disturbances.

Family members were included in the program during the first few days of the first admission as well as during the evaluation stay. If necessary, additional follow-up for participants and family members was provided between the various rehabilitation admissions. Furthermore, the program aimed at establishing good co-operation between the rehabilitation centers and the health care professionals in the participant’s local community, with the aim of securing adequate follow-up after participants completed the in-patient rehabilitation study. There was special emphasis on establishing of an Individual plan. A multidisciplinary team set rehabilitation short and long-term goals together with the participant. Each participant was discussed in a multidisciplinary team during each stay in order to secure optimal rehabilitation for each individual. Further information about description of rehabilitation program is in the Appendix, table A.

A health economical analysis was not built into the evaluation. The program was established with-in the current reimbursement scheme for rehabilitation in Norway, with a daily reimbursement of approximately 3 000 NOK (equals 500 USD) per patient. The cost of a three-week program for each patient will hence be approximately 63 000 NOK (equals 10 500 USD).

**Description of outcome measures**

Motor function

As measures of motor function*, *gait and balance were assessed using the following tests: a) *Timed-up-and-go test (TUG)*: the participant stands up from a chair, walks 3 meters, turns around, walks back and sits down on the chair again while being timed. 21 ^,^ 22 b) *10-Meter Walk Test (10MWT): *the participant is asked to walk 10 meters as fast as possible. A 10-meters walking area has two meters extensions before and after starting and finishing line so that allows participant to start and finish walking smoothly. The time to complete walking 10 meters was recorded and average gait speed was calculated. 22 ^,^ 23 c) *Six Minute Walk Test (6MWT)*: the distance the participant walks within 6 minutes is measured in meters, 23 ^,^ 24 d) *Berg Balance Scale* (BBS), which consists of 14 subtests covering various activities such as static posture, transition, challenging positions, associated with balance control. The quality of performance on each of the 14 tests was recorded using a 4-point scale gaining a maximum score of 56 points. High scores indicate better balance 22 ^,^ 25. e) *Activities of Balance Confidence scale (ABC)*, which is a questionnaire with 16-items describing various tasks for which the participant indicates for each of them how confident they are in performing these tasks without losing their balance or becoming unstable. A higher score indicates higher confidence 26. Assessments a) – d) were completed at the beginning and end of each admission, resulting in a total of seven assessment points. Assessment e) was completed at the beginning of each admission, generating a total of four assessment points.

Activities of Daily Living 

*Barthel index*, a 10-item rating scale, was used to evaluate the level of assistance needed by a participant to perform basic activities of daily living 27. Four items were rated 0-3 or 0-1, and rest of the six items were rated 0-3 for a total maximum score of 20 with higher scores indicating better performance. This test was assessed at the beginning of each admission, a total number of four assessments points.

Cognitive function

*Mini Mental State Examination (MMSE)* was used to evaluate the participants’ general cognitive status. The maximum score is 30 points, with higher score indicating better general cognitive status. A score below 24 is an indication of general cognitive impairment 28. The MMSE was conducted at each admission. The results from the first admission and the evaluation assessment are reported. The *UHDRS Cognitive Assessment*was used to evaluate change in cognitive function from baseline to evaluation stay 20, and includes the following tasks: a) Verbal Fluency Test, requiring the participant to generate as many words as possible beginning with a specific letter (F, A and S) in 60 seconds. The score is the total number of correct words for the three letters. b) Stroop colour-word test, which includes three conditions: naming colour blocks (blue, red or green); reading colour words printed in black ink; naming the ink colour of incongruous colour words (e.g. the word “red” written in green ink). For each condition the score is the number of correct responses produced in 45 seconds. c) Symbol Digit Modalities Test, a paper and pencil task, in which the participant is required to pair digits to assigned symbols using a reference key. The score is the total number of correct written responses in 90 seconds. Higher scores indicate better cognitive performance. The tasks are measures of psychomotor speed and executive function.

Depression and Anxiety

The Hospital Anxiety and Depression Scale (HADS) is a 14 item self-report questionnaire, and was used to assess symptoms of anxiety and depression. Each item is rated 0 – 3, generating a maximum total score of 42 points. Even items from the Depression sub-scale and uneven items from the Anxiety subscale is also possible to rate 29 ^,^ 30. The HADS was administered at the beginning of each admission.

Quality of Life

The Short Form-12 (SF-12) a self-report questionnaire consisting of two component scores for Physical and Mental quality of life, respectively, was used to assess the participants´ quality of life and participation and was assessed at the beginning of each admission 31.

Additionally, the participants’ BMI was assessed during each stay. Participants with a BMI lower than 21 were monitored by a dietitian during the full three-week stay. All assessments were conducted by experienced staff, as far as possible, by the same staff member. The UHDRS motor and cognitive assessments were performed by the same trained professionals (JCF and MvW). All outcome measures used in the present study are widely used in the field of neurological and geriatric rehabilitation.

**Statistical analyses**

For gait and balance variables, the linear mixed effect model of Analysis of Variance (ANOVA) was used to show mean changes from baseline (stay 1) for stay two, stay three and the evaluation stay (stay 4). In the case of a non-normal distribution, non-parametric Friedman’s ANOVA test was used.

For the remaining variables, comparison between baseline and the final evaluation was done using Paired t-test or non-parametric Wilcoxon Signed Rank test depending on the distribution of the data.

The SPSS software, version 20 was used for all statistical analyses. Level of significance was set at p<0.05.

## Results

Characterization of the sample

A total of 37 patients were enrolled in the rehabilitation programs with the following demographic characteristics at baseline: the mean age of the participants was 52.4 (SD±13.1) years and 51.4% (n=19) of the participants were women. 54.1% (n=20) were married and 83.8% (n=31) had children. A minority of participants (24.3%, n=9) were smokers. An Individual Plan was established for 35.1% (n=13) and 44.2 % (n=16) received some kind of assistance at home. Mean symptom duration was 7.2 (SD±5.7) years and mean score for total functional capacity (TFC) was 8.9 (SD±2.3). 24.3% (n=9) participants were in stage I, 56.8 % (n=21) participants in stage II, and 18.9 % (n=7) participants in stage III on Shoulson & Fahn scale. Furthermore, participants had a mean UHDRS motor score of 36.6 (SD±16.7), a mean UHDRS behavioral score of 9.2 (SD±8.5) and MMSE score of 25.4 (SD±3.5). The mean BMI was 22.8 (SD±3.2). Mean time from the first admission to evaluation stay was 377.3 days (SD±55.9) and the mean time from discharge at 3^rd^stay to evaluation stay was 95.4 days (SD ±34.2). Information about patients’ medication use according to disease stage at baseline is in the Appendix, table B.

The patient recruitment was based on physicians’ referrals, and only one patient who was referred did not meet the inclusion criteria. Reason for exclusion was patient’s poor ADL function.

**Effect of Intervention**

Motor function

There was significant improvement in gait (measured by TUG, 10MWT and 6MWT) from baseline through stay two and three to evaluation stay as shown in table 1. The mean change between baseline and evaluation stay in gait assessments were the following: TUG -1.32 seconds, 10MWT –0.27 m/ seconds and 6MWT +68.71 meters. We found that the changes in two gait measures (TUG and 10MWT) exceeded the minimal detectable change values and therefore the changes are clinically meaningful. Our findings support Quinn et al (2013) work^22^ . The appendix, table C, D and E will provide further information.

There was an overall improvement in balance as shown in table 1. The mean change in BBS from baseline to evaluation stay was +1.0 (p<0.03). However, the ABC scale did not show any change from baseline to evaluation stay.

No change was observed in ADL-function as measured by Barthels Index.

**Figure:**
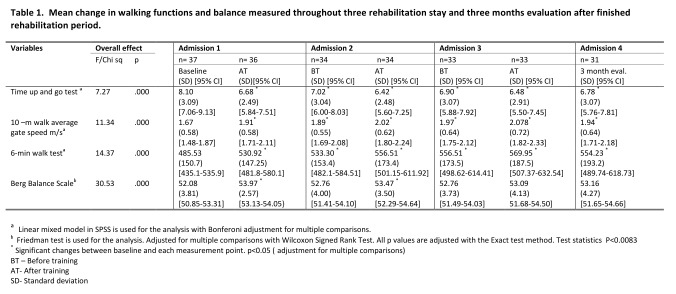

*Cognitive function.* MMSE showed minor improvement of +0.67 points in overall cognitive function. FAS test (cognitive regulation) showed minor improvement in mean score (+0.89). SDMT (psychomotor speed, cognitive effectiveness) showed a significant decline with mean change of 2.87 points (p<0,05). Stroop color naming, and Stroop word reading, both measures for psychomotor (mental) speed showed a minor decline of -1.35 points, and an insignificant increase of 1.50 points, respectively. The Stoop interference test, an executive function task, measuring cognitive inhibition showed a slight decline of -0.04 points. Overall there was no significant change in mean UHDRS cognitive scores among participants during the study period. The results are shown in table 2.

*Anxiety and depression* were significantly reduced (3.54 points) from baseline to the evaluation stay (p<0.001) (table 2).

*Quality of life. *Participants reported significant improvement in the physical component score on the quality of life measurement when comparing baseline to evaluation (table 2). No significant change was found in the mental component score.

Participants gained some weight during the project period, indicated by a change in BMI of 0.72 units (p<0.024) from baseline to evaluation stay.

Finally, we found that a larger proportion had initiated or established a long term coordinated health care plan, *Individual Plan*, at the evaluation stay compared to the start of the program, from 13/37 to 24/33.

**Figure:**
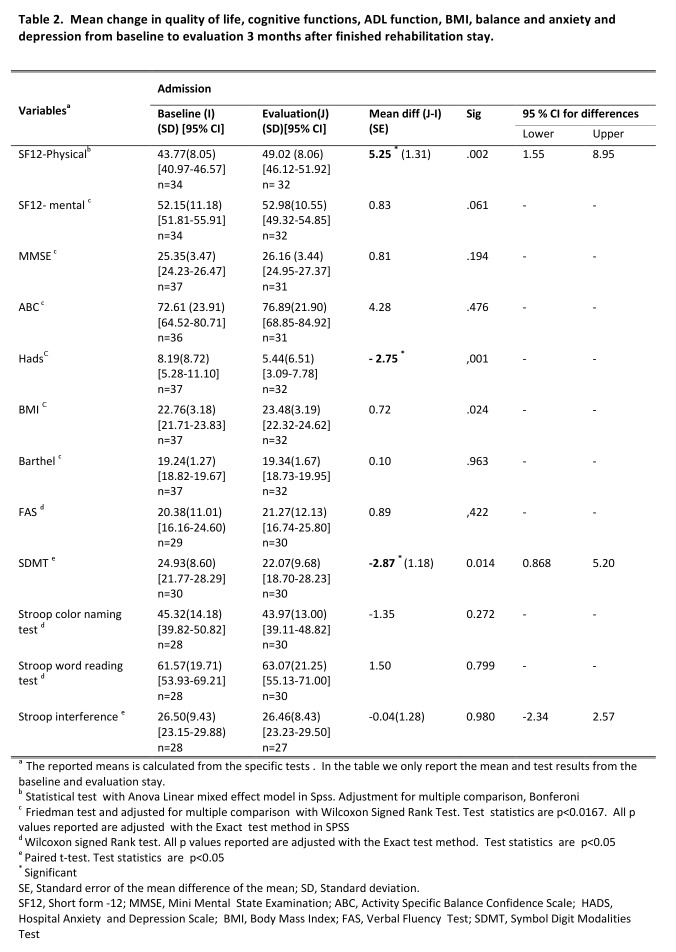

## Discussion

The results of the present study show that participation in a structured intensive multidisciplinary rehabilitation program is associated with improved balance, gait function, physical quality of life and with reduced depressive and anxiety symptoms in patients with early to middle stage HD. Additionally, only one cognitive measure (SDMT) showed significant decline, and no decline was observed for the remaining cognitive measures. These results suggest beneficial effects of an intensive rehabilitation approach on symptom development of early to middle stage HD, and are in keeping with the findings reported by Zinzi et al (2007). Furthermore, adding additional outcome measures (quality of life, UHDRS-cognitive battery and gait assessments), has strengthened previous results in showing that physical quality of life is improved and that specific cognitive domains (psychomotor speed, executive function) overall show no significant decline. Our study accomplished considerably better retention with 31 out of 37 patients (83,8%) completing the full one year program, compared to Zinzi et al (2007) where only 25 of 40 patients (62,5%) completing the third rehabilitation period (first year of the study). Our study demonstrates that HD-patients are able to complete a structured rehabilitation program, and possible explanation for the high retention rate in our study could be planned effort to assist and maintain regular contact with the patients in between stays, and that financial expenses with participating in the program were covered by the health care system. Reasons for drop-out were disease progression, difficulties with transport to / from the rehabilitation facility, reduced ability to be tested due to factors such as severe depression as well as reduced test motivation. We found that an increased number of patients reported having, an Individual Plan, indicating that a larger number of patients have received established long term coordinated health care services.

From the start of the project, it was clear that this was part of patient health care quality assessment and improvement. This implied that after each stay, a comprehensive medical report was sent to the referring physician and other relevant allied health care personnel. The report clearly described the patients’ multidisciplinary needs, with a final medical report sent after completing the entire 1-year program. This may have contributed to a better understanding of the needs of the patients by their local health care personnel.

It is important to be aware of some methodological considerations of the project.**This study is not a randomized clinical trial, but a descriptive study over a period of one year, as part of the evaluation of the implementation of an intensive multidisciplinary rehabilitation program. However, this is still only the second study looking into the effects of a multidisciplinary approach as treatment for HD. And although our study was only a one-year study as compared to the two-year follow-up reported by Zinzi et al., the present study has a better retention rate (31/37 versus Zinzi’s study 25/40). Furthermore, it is important to note that patients in our study received slight adjustments to medication when necessary during the course of the program. This may have contributed/ strengthened the observed beneficial effects of the project.

Strengths of this program include standardized protocols and systematically executed multidisciplinary approach, which had been carefully planned in terms of use of assessments, measurement points, aiming to have the same rater at both the baseline and final evaluation admission. The raters were trained and standardized and were experienced in their field. All patients had received a clinical diagnosis of HD, based on symptoms and known CAG repeat expansion, but we were unable to record the number of CAG repeats for the patients.

Taken together the present study supports previous results from Zinzi et al (2007) that an intensive multidisciplinary rehabilitation approach can be useful in the management and treatment of symptoms of early and middle stage HD. The study also shows the potential for good retention. Furthermore, in addition to functional outcomes, the present study seems to have contributed to increased establishment of long term and coordinated health care delivery for the participant. In Norway the positive effects of the study have resulted in the establishment of permanent rehabilitation services for HD patients. Whether and how long the observed beneficial effects can be sustained needs to be assessed with longer follow-up. Moreover, there is a need for randomized clinical trials to study the effect of multidisciplinary intensive rehabilitation intervention on progression of HD, and it is important to investigate which participants profit most from such intensive rehabilitation. The use of complex and long self-reported rating scales and long assessment batteries should be considered when planning future multidisciplinary rehabilitation studies and programs for patients with HD.

We do not know surely which component of the multidisciplinary approach is the most effective but it seems that the structured physical training/activities will have the greatest effects on patients with HD in early and middle stage. We hope that results from our study can contribute and inform care development for patients with HD. In the future, it would be interesting to investigate whether this type of intervention will result less need for supportive care in long-term since this was not in a scope our study. It would be interested to know if it is possible to gain similar positive results with a shorter intervention program for instance 2 x 3 weeks intervention versus 3 x 3 weeks intervention during 12 months included cost-benefit analysis. Economical evaluation of the intensive rehabilitation program is very interesting question but this was out of scope in our study. Future research is needed to evaluate of cost-benefit of intensive rehabilitation program among HD population with an appropriate study design.

##  Clinical message

- Participation in multidisciplinary rehabilitation program seems to be beneficial for persons in early and middle stage Huntington’s disease.

- A structured multidisciplinary rehabilitation program improves motor function and quality of life.

- It is feasible for persons with HD to complete a yearlong intensive multidisciplinary rehabilitation program

- There should be emphasis to establishment of long term and coordinated health care services for the HD patient

## Conflict of interests

Authors declare no conflict of interests

## Ethics statement

Written informed consent was obtained from each patient in separately in both Rehabilitation sites in Tromsø and Vikersund

## Figures and Tables

**Figure:**
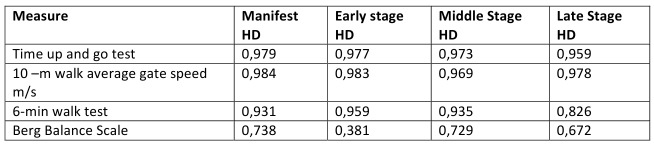
Interclass correlation coefficients were calculated to assess the relative test –retest reliability. The ICC (2. k) was used (the two way random model of consistency), either a type 2, 1 single measure or a type 2. 2 average measure. For 6MWT and BBS the ICC single measure was used while the ICC (2.2) was used for 10 meter walk test and time up and go test because final scores for theses outcomes were based on average of 2 trials.^22^

## Appendix 1

### Table A

**Description of the rehabilitation program for patients with Huntington’s disease**

### Table B

**Medication use according to disease stage at baseline (n=37)**

### Table C

**Mean (SD) 95 % confidence interval [range] and sample size (n) for participants with Manifest HD by stage of disease at baseline measurement**

### Table D

**Intra class correlation (ICC) **

### Table E

**Minimum detectable Change (MDC)** 22 ^,^ 32
